# Mechanism Underlying the Regulation of Mucin Secretion in the Uterus during Pregnancy

**DOI:** 10.3390/ijms242115896

**Published:** 2023-11-02

**Authors:** Mengru Zhou, Tian Tian, Chenchen Wu

**Affiliations:** College of Animal Veterinary Medicine, Northwest A&F University, Yangling 712100, China; zhoumengru@163.com (M.Z.); tiantian@163.com (T.T.)

**Keywords:** uterus, mucins, reproductive hormone, secretory mechanism, glycosylation

## Abstract

The function of endometrial epithelial cells is to secrete various substances that are rich in growth factors and nutrients. These substances support both embryo implantation and its subsequent development into a fetus. A vast number of mucins are expressed in endometrial epithelial cells, and they play an important role in regulating the processes of embryo implantation, pregnancy, and parturition. Previous studies have shown that mucin forms a mucus layer covering endometrial epithelial cells, which helps resist damage from foreign bacteria and their toxins. Therefore, this article aims to investigate the location of mucins in the endometrium, the mechanism of mucin secretion by the endometrium, and the regulation of mucins in the uterine epithelium by reproductive hormones, as well as the role of mucins in the protection of the epithelium’s structure. This research aims to provide a foundational understanding for future studies on the role and mechanism of endometrial mucins throughout the pregnancy cycle.

## 1. Introduction

The uterus is where a fertilized egg develops into an embryo. After fertilization in the oviduct, the oocyte enters the uterus and matures into an embryo [1]. There are mucin O-glycans in both plants and animals, with cytoplasmic membranes, the extracellular matrix, and connective tissues being the main locations of abundant glycosylation [2]. Intracellular glycans need to undergo glycosylation to generate mature mucins. Glycosylation, expressed on the extracellular surface, is connected with cell–cell interactions and the crucial signaling of pathological states. Therefore, O-glycosylation is prominently expressed in the mammalian uterus and is essential for its proper physiological activity. Mucins, which are O-glycosylated macromolecular proteins, are found on the apical surface of polarized uterine epithelial cells. Mucins are involved in the initial stages of embryo and uterus interactions during implantation [3,4,5], and constitute the main component of the mucus layer. The uterine mucus layer must provide a protective function for the tissues while also allowing embryo adhesion. The process of implantation requires an attachable blastocyst and a receptive uterus [6]. During implantation, steroid hormones like estradiol and progesterone modify the mucus layer of the endometrium to make it receptive. These hormones also influence the membrane elements essential for interactions with trophoblast cells [7]. It has been shown that high levels of mucins inhibit cell–cell interactions via ligand access to spatial sites on the cell surface and that the uterus must undergo many different transformations to support fertilization and fetal development; a reduction in or loss of function in the mucosal barrier can lead to endometrial inflammation or infection [8]. Therefore, this review will focus on the location and secretion mechanism of mucin in the endometrium and its regulation by reproductive hormones in uterine epithelial cells. We will also develop the mechanism of attachment for uterine epithelial cells. Our aim is to establish a theoretical foundation for future research on mucin’s secretion, regulation, and protective role in the endometrium.

## 2. Location of Mucin in the Endometrium

**Figure 1:**
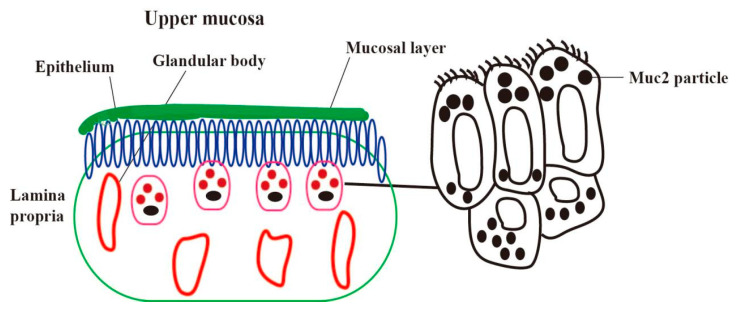
Expression and distribution of mucin in endometrial epithelial cells. The mucin distribution of endometrial cells (red dot, left); there are a large number of glycoprotein particles in the cytoplasm of epithelial cells and basal cells (black square, right).

The uterus plays a pivotal role in reproduction, facilitating the implantation of the embryo and providing a nurturing environment for fetal growth and development. One important function of the uterus is to participate in the implantation of the embryo and provide a place for the growth and development of the fetus, in addition to maintaining the pregnancy until the fetus is delivered [9]. Mucin, a highly O-glycosylated protein, is secreted by goblet cells. Its structure consists of a homologous oligomer with an N-terminal oxidative glycosylation domain and a C-terminal cysteine-rich domain [10]. The mucins include secretory mucins (MUC2, MUC5AC, MUC5B, MUC6, etc.) and transmembrane mucins (MUC1, MUC3A, MUC3B, MUC4, etc.). In our previous experiments, it was found that MUC1, MUC2, and MUC4 were the main mucins expressed on the uterus [11]. Mucin plays a key role in forming the intestinal mucus barrier, its primary defense against pathogenic bacteria and harmful external substances. Changes in the quality and quantity of mucins are related to a variety of intestinal diseases [12]. Mucin is also a major component of the apical surface of uterine epithelial cells, which are involved in the initial stages of embryo–uterine interactions during implantation. While uterine epithelial cells serve a protective function for the tissue, these cells must also allow the embryo to attach [13]. MUC1 (Mucin-1), also known as epithelial mucin, was the first mucin core protein to be cloned from both humans and mice. In a previous study, we observed numerous glycoprotein particles at the apex and base of endometrial epithelial cells. Using immunohistochemistry, we analyzed the distribution of MUC2 (Mucin-2) and detected a positive signal for these glycoprotein particles. This suggests a significant expression of MUC2 in endometrial epithelial cells [14]. When inflammatory factors stimulate the uterine tissue, the expression of the mucous layer at the top of the uterine epithelial cells (MUC2 is the main component) is increased, and, in most cases, these mucins appear to protect the mucosal surface from infection and the effects of degrading enzymes (Figure 1) [13]. 

## 3. Mechanism of the Secretion of Mucin in Endometrial Epithelial Cells

The exocytosis of secretory mucins is the final step in the process in the cell; secretory mucins are released into the uterine lumen to interact with water and ions to form the mucosal layer. The secreted mucins are only produced in gland cells, not in ciliated cells, mucins are synthesized in the rough endoplasmic reticulum and extend into the basilar region of the cytoplasm. These newly synthesized proteins are transported from the endoplasmic reticulum to the Golgi apparatus in coated vesicles, undergoing complete glycosylation as they traverse the Golgi apparatus [11,15,16]. In the trans-Golgi, mucins may be separated from other proteins, processed, or packaged into larger vesicles. Subsequently, these vesicles may fuse to produce large secretory particles [17]. These transport vesicles are released into the extracellular matrix through a process called exocytosis. This membrane fusion is facilitated by fusion proteins. Central to this process is the four-helix bundle core of the secretory cell, known as the SNARE (soluble N-ethylmaleimide-sensitive factor attachment protein receptor) complex, which assembles on the Munc18 scaffold. Its primary function is to mediate fusion between secretory particles and the plasma membrane [18]. The SNARE protein, a major superfamily protein, plays a pivotal role during the fusion phase of vesicle and target membrane transport. Its subtypes include VAMP, syntaxin (stx), and SNAP (synaptosome-associated protein).

VAMP (vesicle-associated membrane protein 8), located on the vesicle membrane, is termed V-SNARE, while syntaxin and SNAP, found on the plasma membrane, are referred to as t-SNARE. These SNARE proteins possess 60-amino acid coiled-coil domains. The interactions between SNARE proteins not only ensure the specificity of binding between adjacent plasma membranes but also supply the energy required for plasma membrane fusion [19,20,21]. Both syntaxin and VAMP proteins have transmembrane domains anchoring them in place, whereas SNAP proteins are tethered to the plasma membrane via palmitoyl groups situated between their two SNARE structural domains.

**Figure 2:**
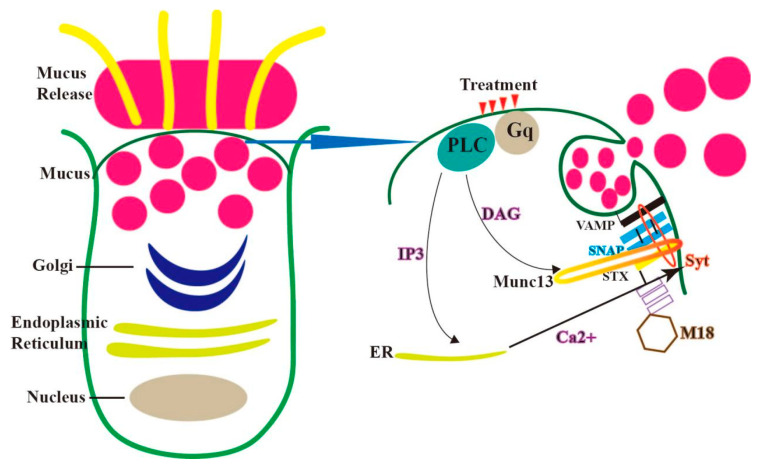
First, the transcribed MUC2 monomer forms a dimer via C-terminal disulfide bonds in the endoplasmic reticulum of goblet cells, for example. The accumulation of unfolded proteins in the endoplasmic reticulum may induce endoplasmic reticulum stress. The MUC2 dimer further completes O-glycosylation in the Golgi apparatus, and the protein core region is protected by polysaccharides, forming a bottlebrush-like structure (**Left**). In a resting state, mucin particles (rose red) bind to the plasma membrane via the interaction between Rab and the promoter Munc13 protein. Agonists bind heptahelical receptors, such as adenosine triphosphate (P2Y2R), adenosine (A3R), and serine protease (protease-activated receptors 1 and 2), first activating trimeric G protein (Gq), and then activating Phospholipase-cβ (PLC). This is followed by the production of second messenger diacylglycerol (DAG) and inositol triphosphate (IP3). DAG activates the promoter Munc13 protein and scaffold protein Munc18 to synergistically open synapsin (Stx), promoting the interaction between Stx and SNARE proteins (synaptic-associated protein (SNAP) and vesicle-associated membrane protein (VAMP)). IP3 induces the release of Ca^2+^ from the apical endoplasmic reticulum (ER), activates the extracellular calcium sensor synapsin (Syt), and promotes the coiling of SNARE complex; the complete coiling of the SNARE complex induces the fusion of particles and the plasma membrane, releasing mucins into extracellular space (**Right**).

Before secretory particles can interact with the plasma membrane, they must traverse cytoskeletal interactions governed by MARCKS proteins (myristoylated alanine-rich C-kinase substrate) [22]. Subsequently, the surface of Rab GTPases on these particles interacts with tethering proteins on the plasma membrane, initiating interactions with cytoplasmic proteins Munc13 and Munc18, which facilitate the opening of synaptic proteins. Rab proteins and Munc13 anchor vesicles to the cell membrane, with SNAP, stx (synaptosome-associated protein), and munc18 (syntaxin-binding protein-18) forming a complex prior to this anchoring. External stimuli generate second messengers DAG and IP3 [23]. While DAG (diacylglycerol) activates Munc13, displacing munc18 and causing stx to adopt a closed structure, IP3 triggers the release of Ca^2+^ from the rough endoplasmic reticulum, activating Syt proteins and promoting SNARE (soluble N-ethylmaleimide sensitive factor attachment protein receptor) intertwining [24]. The initial SNARE protein interactions result in a tight docking between vesicles and the plasma membrane. However, complete coiling and membrane fusion of the SNARE complexes only occur once second messengers, especially calcium and the exocrine calcium sensor synaptic-binding protein (Syt), bind to exocrine cell components, facilitating cellular exocytosis (Figure 2) [22,23]. 

## 4. The Regulation of Mucin in Uterine Epithelial Cells by Reproductive Hormones during the Pregnancy Cycle

Key reproductive hormones, such as estrogen (E2), luteinizing hormone (LH), progesterone (P4), and follicle-stimulating hormone (FSH), play pivotal roles in controlling estrus and pregnancy. These ovarian hormones balance embryo growth with uterine development and oversee processes like ovulation, pregnancy, parturition, and lactation [25]. As levels of FSH and LH increase, they stimulate follicular maturation and estrogen secretion. This prompts the uterine epithelium to enter the proliferative phase. With the maturation of the follicle, estrogen secretion peaks, inducing the hypothalamus to produce a surge in LH and FSH levels, signaling the ovary to begin ovulation [26]. Post-ovulation, the residual follicle transforms into the corpus luteum, which secretes both progesterone and estrogen. This stimulates the endometrium to transition from the proliferative to the secretory phase. If fertilization does not occur, the corpus luteum degenerates, leading to a decline in progesterone and estrogen levels, causing endometrial necrosis and bleeding, marking the onset of the next menstrual cycle [27]. 

If fertilization occurs, the uterus becomes receptive, shifting its dominant regulation from estrogen to progesterone. During the estrogen-dominant period, the uterine epithelium is covered with apical microvilli, and a glycoprotein structure called the glycocalyx forms on its surface. As well as uterine epithelium, there is glycoprotein, which forms a structure called glycocalyx [28]. Generally, estrogen influences the thickness of the glycocalyx, but the nature of these changes varies across species during the implantation period [29]. In certain species, the glycocalyx appears sparse during post-implantation and becomes more compact during implantation. For instance, during days 12 to 18 of the pregnancy period in swine, the glycocalyx thickness increases but decreases post-embryo implantation. Female reproductive hormones function via E2 receptors (ER) and P receptors (PR). While ER may not directly regulate the MUC1 gene in humans or mice, it does regulate MUC1 mRNA in breast cancer cells via ERα. Progesterone maintains elevated levels during the luteal phase, during which MUC1 in the uterus exhibits peak expression. PR (progesterone receptor) has two isoforms, PRA and PRB, which differentially regulate MUC1 gene transcription in response to P4 [14,30]. The P4 reaction zone is situated on the MUC1 promoter at 570/523, which is connected with PRB stimulating promoter activation and protein expression. However, compared to PRB, PRA has the opposite effect on activating the MUC1 promoter. A transactivation function domain (AF3) is responsible for the activation of certain target genes by PRB, and not by PRA, owing to differential binding of co-factors, which, in some cases, have the opposite transcriptional activities [31,32,33]. Specifically, in human uterine epithelial cells, ligand-bound PRB stimulates MUC1 promoter activation, while PRA represses it. PRA can also inhibit the E2-mediated activation of mouse MUC1 expression, suggesting species-specific regulatory differences in MUC1 expression. The opposite effect is caused by the differently regulated expression of MUC1 in some species. Steroid hormone receptor co-regulatory factors further influence the biological role of PR.

The outermost layer of the glycocalyx comprises uterine mucins, primarily MUC1 and MUC2. These lubricating, water-soluble proteins are present on epithelial cell surfaces, offering protection against bacterial attacks and proteolytic enzymes [34]. Extensive glycosylation of MUC1 in the extracellular domain hinders mature embryo implantation and promotes receptor entry by inhibiting cell surface adhesion. In vitro studies have shown that a decrease in MUC1 expression aids in the adhesion of blastocyst secretion factors. During the proestrus and estrus phases, mice exhibit elevated MUC1 expression in the uterus, aligning with E2 fluctuations [35]. While E2 can stimulate mouse MUC1 expression, the effects of P4 and E2 can be antagonistic.

**Figure 3:**
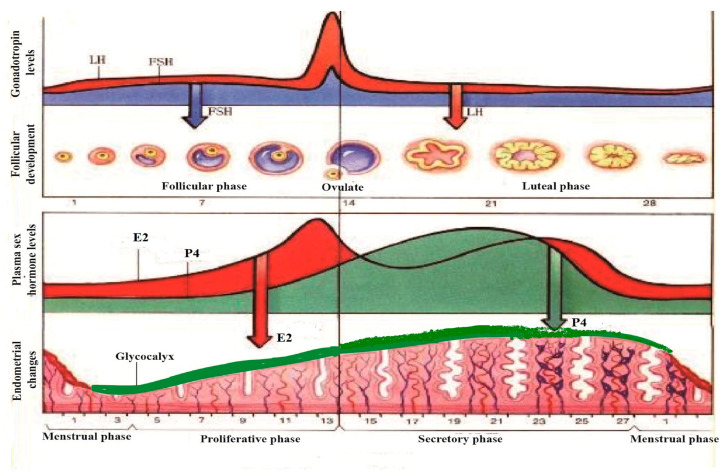
The regulation mechanism of reproductive hormones to ovary and uterine cells. Ovulation after FSH and LH reached peak levels (first line). The estrus reached peak levels; then, FHS and LH reached peak levels after a few hours (second line). The endometrium developed from a proliferative stage to a secretory stage, and the glycocalyx attached to the endometrium increased with P4 excretion. The level of glycocalyx became sparse, but it recovered after the failure of implantation during the implantation period (third line).

Since E2 receptors are unable to directly control MUC1 expression, E2’s regulation of MUC1 is indirect. In the P4-dominated luteal phase and the estrogen-dominated follicular phase, the expression of MUC1 mRNA and protein increased significantly. In vitro experiments have demonstrated that E2 and P4 can significantly up-regulate MUC1 in cultured endometrial cells. However, direct treatment with IFN-ɤ leads to MUC1 down-regulation (Figure 3) [14,30]. Further research is needed to fully understand the hormonal regulation of endometrial mucin.

## 5. Effect of Mucin on Implantation during the Pregnancy Cycle

For successful mammalian reproduction, embryos must implant effectively, and the placenta must facilitate nutrient exchange. This process necessitates a receptive endometrium and the embryo’s development to the blastocyst stage, during which the trophectoderm cells of the blastocyst bind to the uterus’s epithelial cells [36]. Mucin, a transmembrane protein expressed on the apical surface of uterine epithelium, provides defense against microbial infections and enzymatic attacks. This protective layer is further bolstered by glycoconjugates secreted by uterine gland cells, forming a mucous layer [37]. During early implantation, the trophectoderm cells’ outer membrane establishes contact with the uterine epithelium. Various adhesion proteins, including heparan sulfate proteoglycan, integrins, and carbohydrate-binding sites, have been identified on the trophectoderm’s surface. For successful embryo implantation, a reduction in mucin levels seems to be essential [38]. The embryo attaches to the heparin sulfate on the uterine epithelium’s apical surface, involving interactions with various proteins like HIP, HB-EGF, and AR. Studies suggest that the reduction in mucin levels in many species is influenced by ovarian steroid hormones, possibly stimulated by embryonic signals. Additionally, changes in mucin oligosaccharide structures might facilitate binding to oligosaccharide receptors on the blastocyst surface [39]. 

In addition to embryo attachment, reduced mucin levels also increase susceptibility to bacterial infections in the reproductive tract, potentially elevating bacterial invasion risks. MUC2 secreted by endometrial epithelial cells forms a protective mucus barrier against bacterial and microbial invasions [32,33]. During pregnancy, in response to foreign substance invasions, uterine gland cells secrete abundant glycoprotein particles, leading to a significant increase in MUC2 expression. However, this heightened mucin secretion can adversely affect embryo implantation rates, potentially leading to pregnancy failure.

**Figure 4:**
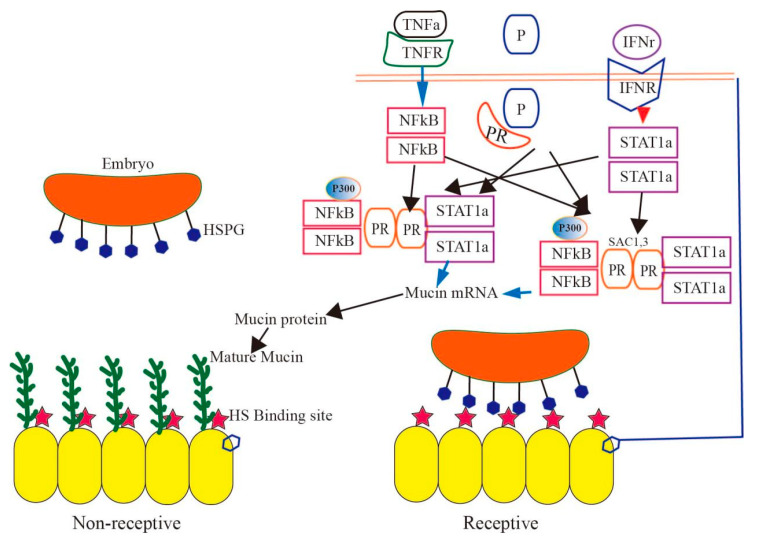
Model of an embryo attached to the uterine epithelium. When the mammalian embryos attach to the surface of uterine epithelial cells, the trophoblast cells around the embryo begin to express cell surfaces that promote attachment. The uterine epithelium is an “unreceptive state”; this state involves the presence of high-molecular-weight mucin that extends into the uterine lumen and provides a spatial barrier for attachment (**Left**). When the uterus transitions into a “receptive state”, mucin is reduced, possibly impacting the steroid hormones and the embryo due to the loss of or reduction in mucin; the embryo can bind to the heparin sulfate (HS) on the surface of the uterine epithelium (**Right**). There are two ways to regulate mucin gene expression through the interaction between the PR subtype and cytokine-activated transcription factors: P, TNF, and IFN are expressed in the uterus during the luteal phase, and pro-inflammatory cytokines (TNF and IFN) bind to their receptors, TNFR and IFNR, to activate the downstream transcription factors NF-κB and STAT1. P binds to its receptor PR, and the PR subtype interacts with NF-κB, STAT1, and transcriptional p300 to regulate the expression of mucin genes. Mucin expression is regulated by NF-κB, STAT1, p300, nuclear SRC-1 and SRC-3, and activated PR subtypes. The interaction of these two pathways increases mucin mRNA and protein expression, and highly glycosylated (mature) mucins reach the plasma membrane (**Top right**).

The function of mucin is mainly determined by the high level of glycosylation of O-glycans [40]. When inflammation occurs in the uterus, the glycosylase of mucin O-glycans is activated, forming a barrier function in the uterine mucus layer, which can prevent the destructive action of the host and intracellular bacteria. Implantation is also characterized as a physiological inflammatory process. Various cytokines and growth factors are released into the fetus–maternal interface, produced by both the endometrium and blastocyst under the influence of steroid hormones. Cytokines like TNF-α (tumor necrosis factor-α) and IFN-γ (Interferon gamma) can activate specific transcription factors, binding to independent mucin promoter sites, and they activate mucin expression through downstream transcription factors, nuclear factor NF-κB (nuclear factor kappa-B), signal transducers, and transcriptional activators [41]. TNF-α stimulates mucin transcription via NF-κB regulation, while IFN-γ, upon receptor binding, activates STAT via JAK kinase. This activated STAT detaches from the receptor, forms a dimer, and enters the nucleus to regulate mucin transcription. When both these factors and P4 co-regulate, NF-KB plays a predominant role in mucin transcription [42]. INF-α and IL-1β and their corresponding receptors bind to activate the SAPK/JNK signaling pathway, while IL-4 (Interleukin-4), IL-6, and IFN-γ activate JAK/STAT to regulate MUC2 expression. On the one hand, the PI3K/Akt signaling pathway can directly impact the NF-κB pathway, causing the release of a large number of inflammatory factors to stimulate the expression of mucin, and NF-κB can also act on some mucin promoters to up-regulate transcription [43,44,45]. On the other hand, mucin secretion can be affected by the activity of glycosyltransferase and changes in glycosylation modification. In addition, it can also regulate glycolysis by participating in the remodeling of the energy metabolism, improving the metabolism and expression of mucin. A variety of cytokines are produced at sites of inflammation or infection; they promote the expression of mucin or related genes, thus protecting mucous in the distal part of the tissue [46]. Since PRA inhibits the PRB stimulation of MUC1 expression, it is unclear whether PRA has a similar inhibitory function on the cytokine activation of MUC1 expression. Since the endometrium of some species is dominated by the PRA subtype, this may impair the P4-mediated mucosal protection of pregnancy reactions. Therefore, low-dose proinflammatory cytokines and PR subtypes have the ability to control mucin gene expression (Figure 4) [41,42]. 

## 6. The Effect of Mucin on the Tight Structure of Uterine Epithelial Cells during Pregnancy

The integrity of the endometrial barrier is crucial for normal uterine function [47]. Mucin plays a pivotal protective role in female reproductive function, shielding the endometrium from enzymatic and microbial attacks. Tight junctions between cells form an epithelial barrier, maintaining cell polarity and inhibiting lipid and protein flow [48]. When the uterus is invaded by foreign entities like bacteria or toxins, the first line of defense is the endometrial mucous layer, which increases its mucin secretion to protect fetal development. An influx of foreign substances can damage this mucous layer, further affecting the endometrial epithelial cells and disrupting the tight junction proteins [49]. The tight junction makes the epithelial cells more closely connected, forming the first line of defense for epithelial contact with the outside world. It plays a key role in maintaining the internal environment and stability of tissues [50]. 

**Figure 5:**
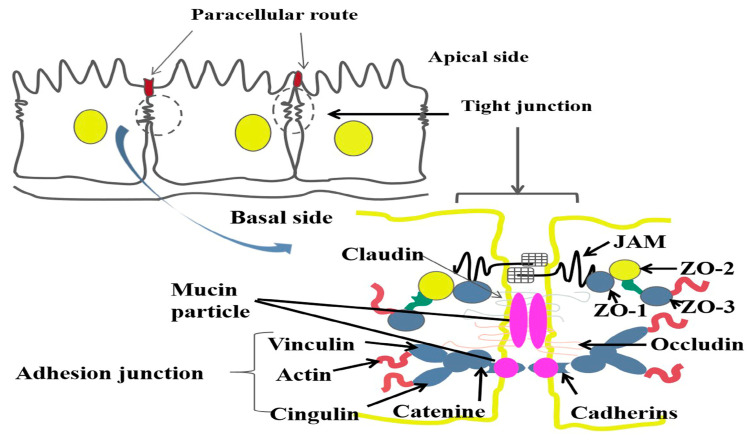
Interaction of endometrial mucin and tight junction proteins during the recovery period. The mucin (pink circle) is attached to the E-cadherin (blue dot) and Claudin (blue dot) of the tight junction in the epithelial cells of the uterine tissue during recovery.

Mucin is a high-molecular-weight glycoprotein, characterized by a core peptide chain and a glycan chain. Secreted by various epithelial cells, it is primarily found in the mucosal epithelium of the respiratory, urinary, and reproductive tracts [51,52,53]. In the uterus, mucins play roles in anti-adhesion, defense, and blastocyst implantation. Their expression on cell surfaces can inhibit cell-cell and cell–matrix adhesion, as well as prevent receptor–ligand interactions from initiating or mediating cell–cell adhesion on spatial sites [54]. MUC1 can inhibit E-cadherin-mediated cell–cell interactions. While there is evidence of E-cadherin interactions between murine trophectoderm and uterine epithelium, its necessity for embryo implantation remains uncertain [55]. MUC1 might also interfere with other adhesion proteins essential for embryo implantation. It is not clear whether the change in glycocalyx thickness is due to the specific function of MUC1. However, in addition to MUC1, other mucins have also been found in the uterine epithelium of mice and humans. Given that other mucins found in the reproductive tract are secreted, and little is known about how they remain relatively stable on the cell surface [56], the question is whether MUC1, as a transmembrane mucin, is anchored to the cell surface of other proteins. In our research, it was observed that the expression of MUC1, MUC2, and MUC4 on the endometrium decreased after the seventh day of pregnancy. After lipopolysaccharide (LPS) treatment, the expression of tight junction-related proteins like Occludin, JAM (junctional adhesion molecule), and E-cadherin in endometrial cells also decreased [57]. On the 14th day of pregnancy, LPS stimulation in mice led to significant reductions in MUC1, MUC2, and MUC4 expressions on the endometrium. Concurrently, while Claudin, JAM, and E-cadherin levels significantly decreased, Occludin expression increased, potentially protecting the endometrial barrier [58,59]. When LPS did not stimulate the endometrium, the damaged uterine tissue began to recover, and mucin was found to attach to the tight junction protein of the epithelial cell and interact with E-cadherin, Claudin, and JAMA to promote the structure of the endometrial epithelial cells (Figure 5) [33]. It remains to be determined whether mucin interacts with the tight junction proteins of epithelial cells to promote the recovery of endometrial tissue.

## 7. Conclusions

Numerous studies have highlighted the significance of O-glycan mucins in the uterine environment in embryo implantation, pregnancy, and parturition. The exocytosis of secretory mucins is only produced in gland cells, which appears to protect the mucosal surface of the uterus from infection and the effects of degrading enzymes. The mucins are released into the uterine lumen to interact with water and ions to form the mucosal layer, which is regulated by reproductive hormones. Specifically, during implantation, the structure of mucin O-glycan undergoes transformation, preparing the endometrium to bind to the oligosaccharide receptors present on the blastocyst’s surface; this process is regulated by many signaling pathways. During pregnancy, the changed structure of mucin O-glycans attaches to endometrial epithelial cells to function as a tight repair structure of uterine epithelial cells when defending against the attack of foreign bacteria and their toxins. However, the functional role of mucin O-glycans has not been clearly defined, and future research in this field will be highly valuable, which may offer insights into improving reproductive health or addressing infertility issues in humans.
